# Planar Proton Minibeam Irradiation Elicits Spatially Confined DNA Damage in a Human Epidermis Model

**DOI:** 10.3390/cancers14061545

**Published:** 2022-03-17

**Authors:** Harry Scherthan, Stephanie-Quinta Wagner, Jan Grundhöfer, Nicole Matejka, Jessica Müller, Steffen Müller, Sarah Rudigkeit, Matthias Sammer, Sarah Schoof, Matthias Port, Judith Reindl

**Affiliations:** 1 Institut für Radiobiologie der Bundeswehr in Verb. mit der Universität Ulm, Neuherbergstr. 11, 80937 München, Germany; stephanie-quinta.wagner@gmx.de (S.-Q.W.); jessica4mueller@bundeswehr.org (J.M.); steffen4mueller@bundeswehr.org (S.M.); sarahschoof@bundeswehr.org (S.S.); matthiasport@bundeswehr.org (M.P.); 2 Angewandte Physik und Messtechnik, Universität der Bundeswehr München, Werner-Heisenberg-Weg 39, 85577 Neubiberg, Germany; nicole.matejka@unibw.de (N.M.); jan.grundhoefer@unibw.de (J.G.); sarah.rudigkeit@unibw.de (S.R.); matthias.sammer@unibw.de (M.S.)

**Keywords:** 53BP1, cell death, DNA damage, epidermis, γ-H2AX, proton minibeam radiation therapy, DNA repair, skin model

## Abstract

**Simple Summary:**

Radiotherapy can lead to severe side effects involving the skin. Proton minibeam radiotherapy (pMBRT) can avoid such side effects by sparing healthy tissue between radiation beams. Here, we spatially mapped DNA damage in response to different widths of proton minibeams (66, 408 and 920 µm) at escalating times after pMBRT, and discovered that focused 66 µm pMBRT induced severe DNA damage at the dose peaks, while damage in the spared tissue was not apparent. Wider proton minibeams (408, 920 µm) damaged all cells. Seventy-two hours after irradiation, DNA damage was repaired, while apoptotic cell death (active caspase-3+) increased significantly 24 and 72 h after pMBRT. Hence, highly focused minibeam-irradiation spares healthy tissue from DNA damage and cell death, which may add to the tissue-sparing effect observed at the macro scale. Thus, highly focused pMBRT may be used in radiotherapy to reduce side effects to the skin.

**Abstract:**

*Purpose*: High doses of ionizing radiation in radiotherapy can elicit undesirable side effects to the skin. Proton minibeam radiotherapy (pMBRT) may circumvent such limitations due to tissue-sparing effects observed at the macro scale. Here, we mapped DNA damage dynamics in a 3D tissue context at the sub-cellular level. *Methods:* Epidermis models were irradiated with planar proton minibeams of 66 µm, 408 µm and 920 µm widths and inter-beam-distances of 2.5 mm at an average dose of 2 Gy using the scanning-ion-microscope SNAKE in Garching, GER. γ-H2AX + 53BP1 and cleaved-caspase-3 immunostaining revealed dsDNA damage and cell death, respectively, in time courses from 0.5 to 72 h after irradiation. *Results:* Focused 66 µm pMBRT induced sharply localized severe DNA damage (pan-γ-H2AX) in cells at the dose peaks, while damage in the dose valleys was similar to sham control. pMBRT with 408 µm and 920 µm minibeams induced DSB foci in all cells. At 72 h after irradiation, DNA damage had reached sham levels, indicating successful DNA repair. Increased frequencies of active-caspase-3 and pan-γ-H2AX-positive cells revealed incipient cell death at late time points. *Conclusions*: The spatially confined distribution of DNA damage appears to underlie the tissue-sparing effect after focused pMBRT. Thus, pMBRT may be the method of choice in radiotherapy to reduce side effects to the skin.

## 1. Introduction

Dose escalation in radiotherapy is restrained by the adverse side effects in normal tissue, especially the skin [1], which in turn limits the dose to the tumor volume. Such restrictions may be circumvented by spatial fractionation of radiation as done in X-ray microbeam radiotherapy [2,3,4] or proton minibeam radiotherapy (pMBRT) [5,6]. The aim of pMBRT is to minimize normal tissue injuries in the entrance region and especially to the skin, which is often a limiting organ [7], by interdigitating small irradiated regions with non-irradiated ones. To do so, the radiation is applied in beams with a size of approximately 50 µm up to 1 mm and with distances of several millimeters [8]. The tissue lying in the path of the focused beams is exposed to a high acute dose, whereas tissue between the beams is spared from radiation. Below the surface area, the proton minibeams widen due to the Coulomb scattering of the protons at molecules in the tissue. This generates a more homogeneous dose distribution in the tumor volume that resembles conventional broad beam radiotherapy, an effect that will contribute to tumor control. So far, tissue-sparing by pMBRT has been demonstrated in a mouse ear model [9,10,11] and also in rat brain [12,13,14]. These investigations showed that the application of minibeam geometry spares healthy tissue, as indicated by reduced inflammation and fibrosis as well as brain lesions. In agreement, a reduced inflammatory response and higher viability were noted in a human skin model, compared to homogeneous broad-beam irradiation [6]. Furthermore, in a rat glioma model, it could be shown that pMBRT increases survival rate by keeping tumor control [12,13,14]. The sparing of healthy tissue is attributed to two effects, the so-called dose-volume effect and the microscopic prompt tissue repair (reviewed in [8]). The latter describing the influence of small blood vessels on accelerated tissue repair, which has so far only been studied in animal models. In contrast, the dose-volume effect describes an increase of the maximum tolerated dose upon decreasing irradiated tissue volume [15] based on the infiltration of healthy cells from the non-irradiated regions into the irradiated and damaged volume [16]. It is also assumed that bystander and rescue effects on the single-cell level might play a role in the damage response [17,18]. Understanding the basic mechanisms of the tissue-sparing effect of spatial fractionation is of crucial importance to find optimal irradiation (IR) settings for clinical use. However, the damage inflicted by pMBRT at the single-cell level in a 3D tissue context has so far not been demonstrated.

The aim of this work was to elucidate the damage distribution in a human epidermis tissue model irradiated with proton minibeams in comparison to broad beam irradiation using the ion microprobe SNAKE at the Munich 14 MV tandem accelerator in Garching, GER [19]. Here, we performed IR experiments using planar proton minibeams with beam sizes (σ) of 66 µm, 408 µm and 920 µm and inter-beam center-to-center distances of 2500 µm on epiCS human reconstructed epidermis models that adequately recapitulate the epidermis response to environmental stresses [20,21]. Of the irradiation geometries used, the focused 66 µm planar proton minibeams expose only about 17% of the cells in skin tissue, but with a 26-fold escalated dose in the peaks relative to the 920 µm homogeneous irradiation with 2 Gy.

Since skin tissue models display a potent DNA damage response [22,23,24,25], we studied the induced DNA double-strand break (DSB) damage by 53BP1 and γ-H2AX immunostaining in paraffin cross-sections of epidermis models exposed to the three pMBRT irradiation geometries noted above. DSB damage and its repair, as well as apoptosis induction, were studied in time courses up to 72 h post-irradiation.

## 2. Materials and Methods

### 2.1. Reconstructed Human Epidermis Model

Human reconstructed epidermis (epiCS^®^) models were obtained from CellSystems (Troisdorf, Germany (now Phenion.com, GER)). EpiCS is a certified model for the testing of chemicals [21] and represent a 3D human epidermis reconstructed with normal primary human epidermal keratinocytes by an air–liquid interface culture on a polycarbonate membrane. The 3D structure resembles a fully differentiated natural epidermis with a base membrane, proliferating keratinocytes and a stratum corneum. The model grows on an 8 mm diameter cell culture insert on a semipermeable polycarbonate membrane (Thermo Fisher Scientific Nunc, Langenselbold, Germany) while maintaining an intact barrier function (CellSystems, Troisdorf, Germany). 

After the models arrived at the laboratory, they were placed in six-well plates with epiCS culture medium and cultured at 37 °C overnight for up to three days prior to irradiation (depending on beam access), according to the instructions of the supplier (CellSystems, Troisdorf, Germany). For proton minibeam irradiation, cells were transferred to the MLL tandem accelerator lab in Garching, GER, and prepared as described in detail previously [26]. The orientation of the beams was marked on the models by punching 0.4 mm syringe needle holes asymmetrically into the culture membranes of the inserts prior to irradiation. The inserts were then placed in specially designed sample holders. The holders clamped the insert at a defined position, and the front and back sides were covered by a 6 µm thick Mylar foil, which ensured that sterility was maintained and the sample would not dry out during irradiation. The foil was also thin enough so that the beams would not significantly widen and ions could be detected behind the sample [19,27].

### 2.2. Irradiation and Dosimetry

**Figure 1:**
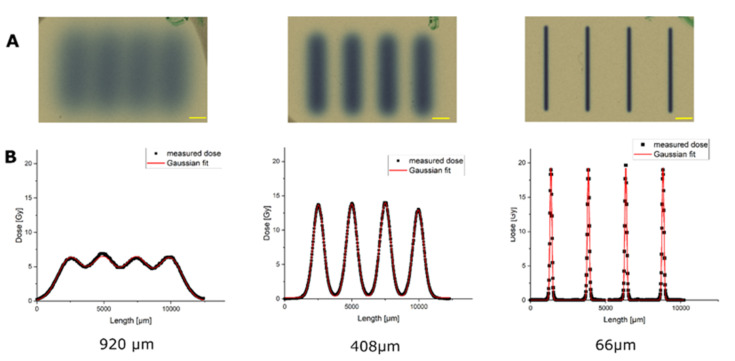
Dose distributions of the three planar minibeam irradiation modes, measured with radiochromic film. Scale bars: 1 mm. (**A**) Gafchromic films after irradiation with the planar beams in three different beam geometries. (**B**) Fitting of the dose distribution of the irradiated geometries along the centerline perpendicular to the proton beams, revealing the different geometries of the pMBRT-irradiated areas. Note that the doses applied here were adapted to the dynamic range of gafchromic films and are therefore different from the tissue irradiation doses of Figure 2.

Focused proton minibeams were generated by magnetic focusing with the ion microprobe SNAKE (Supraleitendes Nanoskop für angewandte kernphysikalische Experimente = superconducting nanoprobe for nuclear physics experiments) at the 14 MV MLL tandem accelerator in Garching, GER, at which cell nuclei can be irradiated with counted protons, thus allowing a precise dose application. During irradiation, the skin models were retained in a specially designed container [6] and mounted directly behind the beam exit nozzle, with the dermis facing the beam. For an exact dose calculation, every proton was detected in a scintillator-photomultiplier detector after traversing the sample, with an LET value of 2.66 keV/mm at the position of the epidermis sample. Epidermis models were irradiated with 20 MeV protons with a mean dose of 2 Gy over the irradiated area at the biological end-station. The end-station consists of an inverted Zeiss Axiovert 200 M microscope for ion detection. Due to the horizontal beam application, the samples were irradiated in an upright position for 5–10 min with four parallel lines of different widths, with the lines having a length (in the y-direction) of 5 mm and a center-to-center distance of 2.5 mm (Figure 1). The proton beam was focused on the smallest possible size in the x-direction via optical detection through a scintillator placed on the SNAKE end-station microscope. After beam preparation, an aluminum pad of 200 µm thickness was mounted directly at the beam exit nozzle, as previously described [28]. Scattering of the protons in the pad resulted in an angular spread of the beam with a nearly Gaussian angle distribution in the x-direction and a divergent beam. By changing the distance of the sample to the exit nozzle, the beam size could be varied. For larger beam sizes, the sample was moved away from the exit nozzle. The distance was varied from 1.76 mm for the smallest beam to 25 mm for the mid-sized beam and 61.2 mm for the largest beam width. The minibeam sizes were verified by measurements using radiochromic Gafchrom EBT3 films (Ashland.com, Wiomington, DE, USA) (Figure 1). All sizes were measured in air at the position where the tissue surface was located during the experiment. For size determination, irradiation was performed with doses to fit the dynamic region of the gafchromic film in all cases. Single planar beams, as well as all four beams with center-to-center distances of 2.5 mm for all beam sizes, were irradiated. The dosimetry on the beams was performed as previously described [28,29]. The dose profiles were fitted at 10 locations along the planar beams, and the mean beam size was determined. The three different beam sizes were σ 66 ± 1 µm, 408 ± 5 µm and 920 ± 20 µm as calculated using Matlab. The 920 µm beams were considered to have quasi-homogeneous irradiation geometry. Additionally, sham irradiation experiments were carried out as controls. All experiments were performed in triplicate. Ion detection was conducted using a photomultiplier detector behind the sample, which is connected to an ion counting unit and an ultra-fast beam switch. This allows the irradiation of a counted number of ions per beam and thus a precisely defined dose. The number of ions necessary to apply a mean dose of D = 2 Gy on a defined area can be calculated as $N=\frac{D\rho A}{\mathrm{LET}}$. $\rho =1\;\frac{g}{{\mathrm{cm}}^{3}}$ is the density of the target material, which can be considered to be equal to water for a biological sample. $\mathrm{LET}=2.6\;\frac{\mathrm{keV}}{\mu m}$ is the linear energy transfer of the 20 MeV protons. The irradiated area is the area of the unit cell of a single beam. For planar beams, the unit cell is defined as the length of the beam, which was 5 mm in this case, and the center-to-center distance of two beams, i.e., 2.5 mm here. Therefore, the area $A=2.5\;\mathrm{mm}\;\times 5\;\mathrm{mm}=12.5{\;\mathrm{mm}}^{2}.$ The number of ions in a unit cell, therefore, is calculated as $N=\frac{2\mathrm{Gy}\cdot 1\;\frac{g}{{\mathrm{cm}}^{3}}\;12.5{\;\mathrm{mm}}^{2}}{2.6\;\frac{\mathrm{keV}}{\;\mu m}}=610,531,562$. The calculated dose was verified by Gafchromic film measurements. The sham-irradiated samples were placed at the radiation position for 10 min, corresponding to the irradiation time of the irradiated samples, with no beam at the sample.

### 2.3. Tissue Processing

After irradiation, epiCS models were returned to the incubator at 37 °C and 5% CO_2_ and cultured for different repair times, i.e., 0.5, 6, 24, 72 h. Thereafter, the epidermis models were instantly fixed by replacing the culture medium with 4% acid-free formaldehyde in PBS for 20 min, followed by a 5-min wash in PBS, 0.1% Glycin. Fixed epidermides samples were cut out of the cell culture inserts with their supporting membrane using an 8 mm biopsy punch (Thermo Fisher Scientific, Langenselbold, Germany) and embedded flat in low-melt agarose. Models were then processed for paraffin embedding using a Leica ASP300S tissue processor (Leica Biosystems, Nußloch, Germany). After dehydration in a graded alcohol series and embedding in paraffin, the skin membranes were spatially fixed in molds, such that the resulting paraffin blocks could be cut with the irradiation planes lying perpendicular to the orientation of the microtome knife. 

### 2.4. Immunofluorescence Staining (IF)

Paraffin tissue sections of the epidermis models were cut using a Leica RM2255 microtome at 5 µm for DSB foci and 7 µm for active Caspase 3 analysis, mounted on super-frost plus slides (Carl Roth, Karlsruhe, Germany) and dried overnight at 37 °C. Sections were dewaxed in xylene and rehydrated in a graded series of alcohols. Antigen retrieval was performed by submerging the slides for 40 min in 0.01 M sodium citrate (pH 5) at 95 °C followed by gradual cooling to ~37 °C. Nonspecific protein binding was blocked for 10 min at 37 °C using an in-house developed TCTG buffer (10 mM TRIS, 5% Na-Casein, 0.1% Tween20, 0.1% fish gelatin, pH 7.4). The slides were incubated with the primary antibodies against γ-H2AX (mouse anti-γ-H2AX; Merck Chemicals, Darmstadt, GER), rabbit anti-active Caspase 3 (Abcam, Cambridge, UK; ab13847, Lot No. 3713887), rabbit anti-53BP1 (NB 100-304, Novus Biotechne, Wiesbaden, Germany), for 1.5 h at 37 °C in TCTG buffer, followed by 3 × 5 min washes in TCTG and incubation with the secondary dk-anti-rabbit-Cy3 and gt-anti-mouse-Cy5 antibodies (both Jackson laboratories) for 1 h at 37 °C. Finally, slides were washed 3 × 3 min in TCTG, drained and counter-stained for DNA with 0.2 µg/mL DAPI (4′,6-diamidino-2-phenylindole; Carl Roth, Karlsruhe, Germany) in PBS for 2 min and mounted in ProLong Glass antifade (Thermo Fisher Scientific, Langenselbold, Germany). Near-infrared Cy5 immunostaining was used to eliminate repeated problems with growing green autofluorescence of the support membrane of the epiCS models during fluorescence microscopic analysis.

### 2.5. Microscopy and Quantitative Image Analysis

Fluorescence images of the epidermis model specimens were acquired using the TissueFAXS digital pathology analysis platform (TissueGnostics, Vienna, Austria). For quantitative analysis, the tissue cross-sections were scanned and acquired as digital grayscale images in three channels with appropriate filter settings for Cy3, Cy5 and DAPI using a 40× lens on an Axio Observer Z1 fluorescence microscope (Zeiss, Oberkochen, Germany). Cell nuclei in the tissue sections were identified by software-generated contour masks of DAPI-stained nuclei. DSB damage foci and cells showing pan-nuclear γ-H2AX signals were quantified using the TissueQuest analysis software package (TissueGnostics, Vienna, Austria) with cut-off values determined interactively relative to negative-staining regions in the same section. DSB damage foci in single cells were quantified in the nuclear contour masks using the StrataQuest software package (TissueGnostics, Vienna, Austria). The principle of the methods and the algorithms used can be accessed elsewhere [30]. Position annotation along the epidermis cross-sections was done using StrataQuest algorithms that assigned position tags to each individual nucleus along a cross-section. In this way, DSB foci and fluorescence intensities could be mapped at subcellular spatial resolutions allowing position analysis of DNA damage distribution across various epidermis models. In cases where epidermis cross-sections were curved, the tissue sections were digitally straightened by software-assisted linear alignment (TissueGnostics, Vienna, Austria). For this purpose, the starting point was specified by drawing an annotation mark at the left end of the tissue section. All cell nuclei were then mapped relative to this starting point and finally linearly projected in 2D around the center of the section using an appropriate tissue processing classifier (TissueGnostics, Vienna, Austria).

Active Caspase3-positive cells were interactively enumerated using the Metafer 4 imaging system for slide scanning equipped with VSlide und VSViewer software packages (MetaSystems, Altlussheim, Germany). Since apoptotic cells did not display spatial patterning, the values are expressed as average per model.

Three individual models were irradiated independently for each radiation geometry and time point and analyzed. Three sham controls were performed for each condition and time point. For each model, three consecutive cross-sections were analyzed for DNA damage type and distribution, and for expression of active-caspase-3, and the results expressed as an average and SD. On average, we analyzed 1483 cells per tissue cross-section (±236 SD).

### 2.6. Statistical Analysis

Statistical comparisons were conducted by ANOVA followed by Tukey’s multiple comparison test using Prism (GraphPad Software LTT, La Jolla, CA, USA).

## 3. Results

### 3.1. Dose Distributions

The dimensions of the proton minibeams of SNAKE were verified by using gafchromic films, as shown in Figure 1. To get an optimal signal on the film for size measurements, irradiation of the gafchromic films was performed with doses that matched the dynamic range of the film. It should be noted that the doses for the Gafchrome irradiations did not correspond to the doses applied to the skin models later on. The Gafchrome experiments revealed beam widths of σ 66 ± 1 µm, 408 ± 5 µm and 920 ± 20 µm and beam distance of center-to-center distances of 2500 ± 10 µm. These geometries were used to irradiate the epidermis models.

**Figure 2:**
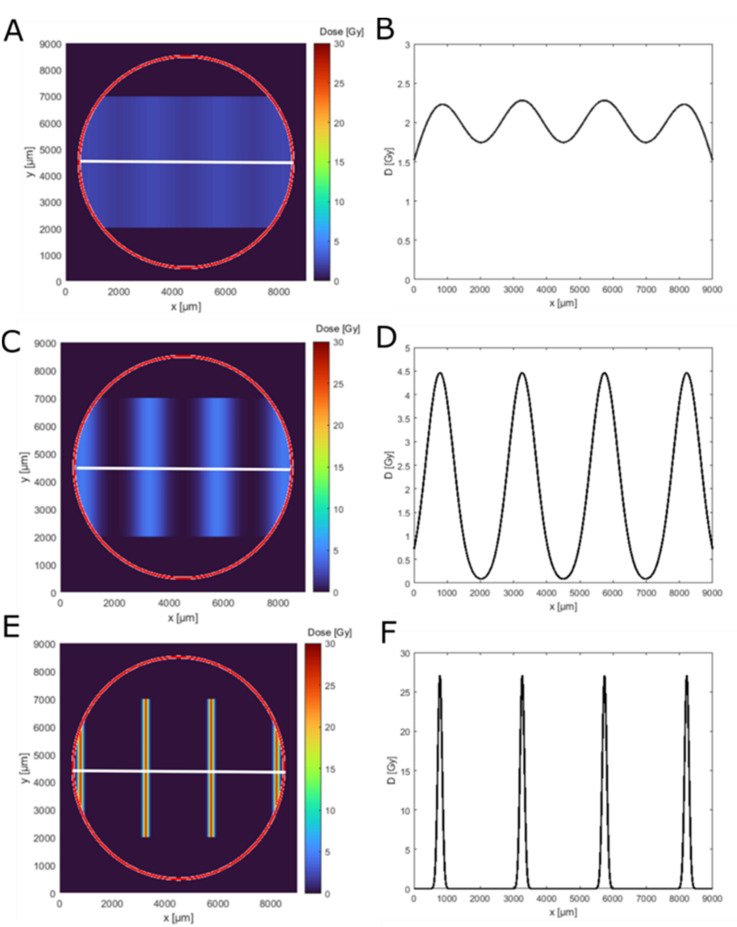
Dose simulation for the used minibeam irradiation geometries on an epidermis model. Panels (**A**,**C**,**E**) show the 2D dose distribution (top view) in the epidermis model insert outline (red circle) for 920 µm, 408 µm and 66 µm beam width. Panels (**B**,**D**,**F**) show the corresponding simulated dose distribution along the white line in the top view (**A**,**C**,**E**).

Next, we computed the dose distribution along the geometry of the skin insert by using the measured beam sizes, the center-to-center distances, the applied mean doses of 2 Gy and the model holder geometry. Figure 2 shows the calculated 2D dose distribution on the epiCS sample for the three beam sizes and perpendicularly along a centerline (Figure 2B). These distributions were later used to interpret the experimental results. The homogeneity of a dose distribution was quantified by the so-called peak-to-valley dose ratio (PVDR): $\mathrm{PVDR}=\frac{D_{\mathrm{Peak}}}{D_{\mathrm{valley}}}$, where $D_{\mathrm{peak}}$ describes the dose in the maximum and $D_{\mathrm{valley}}$ the minimum dose. The closer this factor is to approaching 1, the more homogeneous the irradiation is; the larger the value, the more spatial fractionation is achieved. For the 920 µm beam size (Figure 2A,B), a quasi-homogeneous dose distribution was achieved with a ${\mathrm{PVDR}}_{920}=1.35$ with a peak dose of $D_{\mathrm{peak}}=2.3\;\mathrm{Gy}$. For the middle beam size of 408 µm, all cells of a model are irradiated but with a highly modulated dose with ${\mathrm{PVDR}}_{408}=45$. The peak dose, in this case, was $D_{\mathrm{peak}}=4.5\;\mathrm{Gy}$. For the smallest beam size, the ${\mathrm{PVDR}}_{66}\to \infty$, as only 17% of the cells get irradiated with a peak dose of $D_{\mathrm{peak}}=27\;\mathrm{Gy}$, while the valley dose was computed to be $D_{\mathrm{valley}}=0$ Gy (Figure 2F). The peak dose of 27 Gy was therefore 13.5 times higher than the applied mean dose and more than 10 times higher than the peak dose in the wide-field irradiation. 

### 3.2. Narrow Minibeams Induce Spatially Limited Damage 

**Figure 3:**
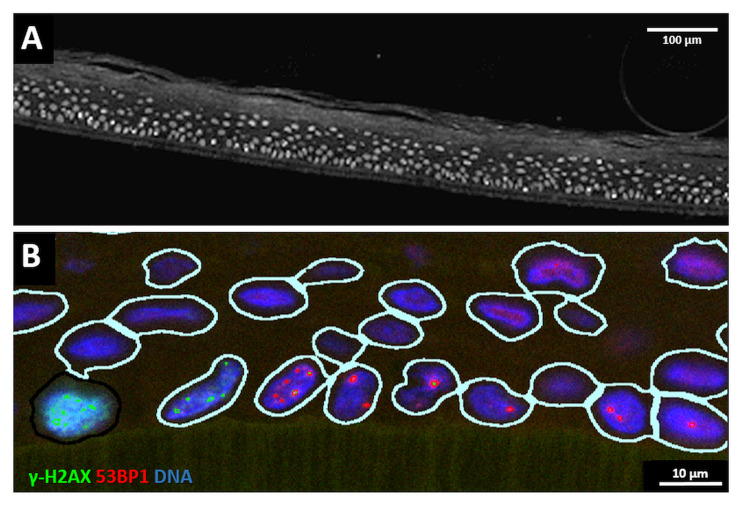
(**A**) Grayscale overview of a part of a cross-section of an epidermis model showing the DAPI-stained nuclei (greyish). The support membrane of the model is at the lower end; above it, the cells of the stratum basale are located, and the keratin layer of the stratum corneum is on top of the cell layers towards the top of the image. Bar: 100 µm. (**B**) Example of nuclei identification and DNA damage analysis in a cross-section detail. The support membrane of the model is at the lower end. Nuclear contour masks (light lines) are generated around DAPI-stained nuclei (blue), 53BP1 DSB foci signals are displayed in red and γ-H2AX signals in green. The cell to the lower-left exhibits a nuclear pan-γ-H2AX signal. Bar: 10 µm. For grayscale images of red and green channels, see Supplementary Figure S2.

First, we determined the DNA damage present in sham-irradiated epidermis models and models exposed to the three proton irradiation geometries. To detect DNA damage in our epidermis models, the orientation of the epiCS models was marked with needle punches prior to proton minibeam irradiation to define the axis of the planar minibeams. After exposure, models were returned to the incubator, followed by culturing for escalating repair times of 0.5, 6, 24 and 72 h. After processing and paraffin embedding, epidermis models were cross-sectioned such that the planar minibeam lines were perpendicularly cut; however, slight variations were unavoidable. After antigen retrieval, sections were immunostained and subjected to automated fluorescence image recording and analysis. Contours of DAPI-stained nuclei in the image data were used to generate masks in which the DNA damage was determined as DSB foci (53BP1, γ-H2AX) or pan nuclear staining (pan-γ-H2AX) (Figure 3). Individual nuclei in the tissue cross-sections were then spatially annotated and mapped for further analysis.

**Figure 4:**
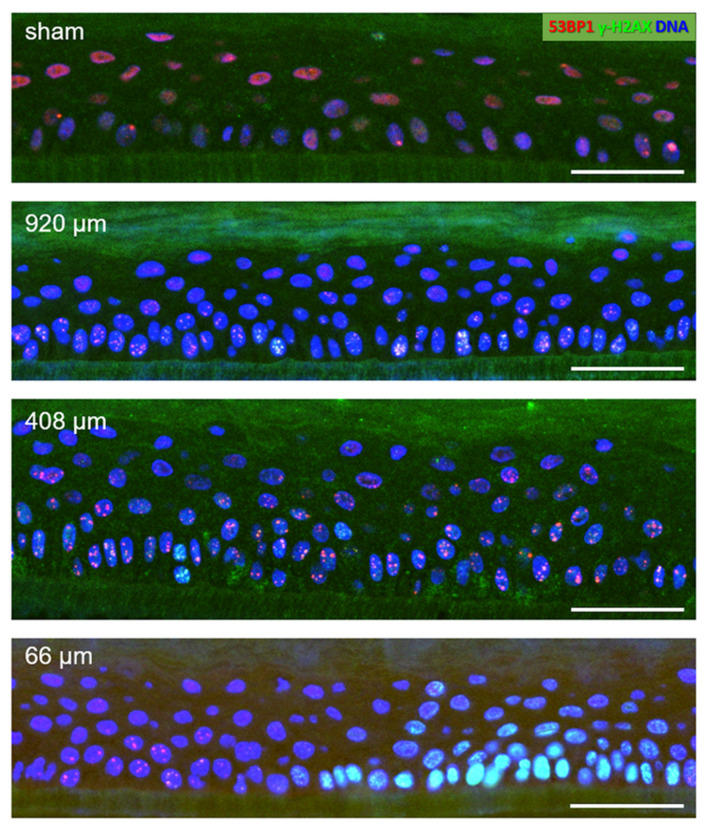
DNA damage in details of epidermis model cross-sections 6 h post-pMBRT. Sham-irradiated epidermis model: few nuclei (DAPI-stained blue) display one or two large 53BP1 foci (red) in the basal layer cells located on the support membrane (dark greenish layer) at the bottom of the images. The reddish diffuse nuclear 53BP1 signal stems from dispersed protein in nuclei without DSBs. 920 µm minibeam irradiated model: Numerous cells showing colocalizing red 53BP1 + green γ-H2AX nuclear DSB foci (yellowish), which is particularly evident in the cells of the basal layer at the bottom of the image. 408 µm minibeam irradiated model: Numerous cells showing colocalizing 53BP1 + γ-H2AX DSB foci. 66 µm minibeam irradiated model: Green nuclei (pan-nuclear γ-H2AX staining) in the right part of the model result from high DSB load in cells hit by the center of the focused 66 µm minibeam. γ-H2AX + 53BP1 DSB foci are seen to the left of the pan-γ-H2AX-positive cell region where the radiation doses fall off steeply. Bars represent 50 µm. For grayscale images of red and green channels, see Supplementary Figure S3.

Sham-irradiated epidermis models usually displayed a low number of cells at or near the basal layer showing one or two large 53BP1 foci (Figure 4). After 920 µm wide-field irradiation, most nuclei carried 53BP1 foci, some showing colocalization with γ-H2AX (Figure 4). The damage load of the cells was increased by 408 µm pMBRT, with some cells displaying a pan nuclear γ-H2AX distribution indicating a high damage load (Figure 4). Models irradiated with the 66 µm proton minibeam showed clustered nuclei with pan-nuclear γ-H2AX staining at the peak dose regions, while cells with distinct γ-H2AX and 53BP1 DSB foci were seen at the edge of the 66 µm minibeam (Figure 4), reflecting the sharply falling off radiation doses from the peak dose of about 27 Gy (Figure 2).

Next, we analyzed the percentage of cells with DNA damage, i.e., at least more than one 53BP1 DSB focus per cell, in the different irradiation conditions and over time. We noted that 53BP1 performed as the most intense marker for DSB-indicating foci in keratinocytes of the models under all conditions. γ-H2AX, in contrast, showed foci of strong intensity only in the basal cell layer cells or at higher doses. At 72 h, γ-H2AX staining created a high background in all models, which precluded its analysis at that time point. Cells along the focus of the 66 µm minibeams displayed a strong pan-γ-H2AX nuclear signal that related to massive DSB load after exposure to more than ~4 Gy (Figure 2D,F) of proton radiation, since pan-γ-H2AX cells were rarely seen in early time points of the wider irradiation geometries (Figure 4). Cell nuclei with pan-γ-H2AX nuclear staining early after irradiation (0.5, 6 h) resulted from the confluence of the H2AX-phospho mark induced by the numerous DSBs in a heavily exposed nucleus, as 27 Gy of low LET irradiation creates approximately 1000 DSBs in a human cell nucleus [31]. In agreement, a pan-γ-H2AX pattern has also been observed in the 50 Gy γ-irradiated minipig epidermis [32] and in cells exposed to high LET irradiation [33]. In our tissue models, pan-γ-H2AX nuclei were also/again seen in active caspase 3 cells and indicated nuclear DNA fragmentation during apoptosis (see below).

**Figure 5:**
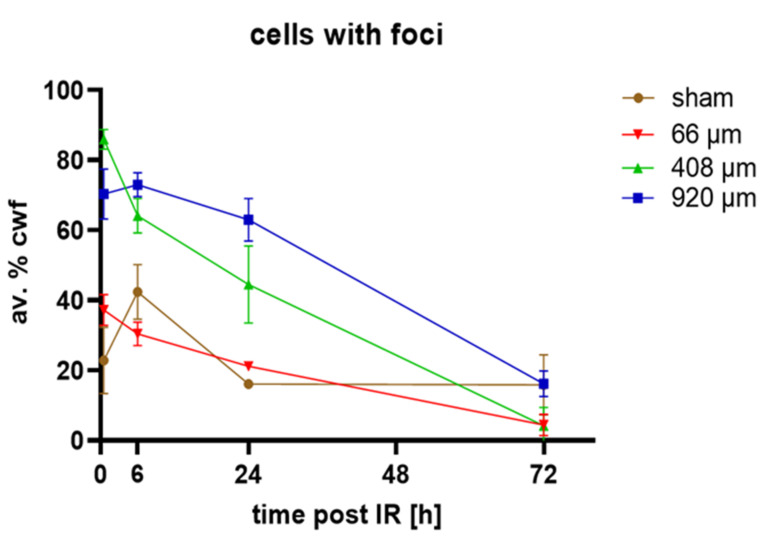
Average percentages of cells with DSB foci (av.% cwf; 53BP1 and/or γ-H2AX foci). Sham-irradiated (brown line) epidermis models showed ~20% cells with foci 0.5, 24 and 72 h post-irradiation, while at 6 h there were 40% cwf. Wide-field (920 µm, blue line), 408 µm (green line) and 66 µm pBMRT induced a significantly increased percentage of cells with foci relative to sham at 0.5 h post-IR. Minibeams of 920 and 408 µm induced significantly increased frequencies of cells with foci 6 h and 24 h after IR. The frequencies of damaged cells were similar to sham at 72 h post-IR. All average values at 72 h represent 53BP1 foci only as there was a large cytoplasmic background for γ-H2AX signals. Each data point represents the average ± SD of *n* = 9 sections of 3 models for each condition, except for 24 h sham (*n* = 6) and irradiated models at 72 h (*n* = 6).

About 20% of cells in sham-irradiated epiCS models displayed 53BP1 DSB foci, which was the case 0.5, 24 and 72 h post-sham irradiation. At 6 h post-sham IR, the average frequency of cells with foci was significantly increased to approximately 40% (Figure 5) in all investigated models (*n* = 3/time point and IR geometry), which may reflect technical issues during transport and handling of these models. Test experiments, for instance, revealed that shear forces prior to fixation can induce caspase 3 activation.

Wide-field 920 µm and 408 µm proton minibeam irradiation created significantly elevated DSB damage in 70% and ~90% of nuclei in the epidermis models, respectively. With increasing repair time there was a gradual decline of cells with DSB foci after 6 h in the wide-field irradiated models (Figure 5). The 408 µm proton-irradiated models showed DNA repair to commence 0.5 h after irradiation (Figure 5). In contrast, models irradiated with the focused 66 µm minibeam displayed a doubling of the average amount of cells with DSB foci 0.5 h post-irradiation, while the average number of cells with DSB foci were similar to sham 6–72 h post-irradiation (Figure 5). Next, we determined the response of the different markers used in our assay within the damaged cells and determined the average amount of DSB-indicating γ-H2AX and/or 53BP1 foci in cells carrying at least one DSB focus. This revealed that pMBRT significantly increased cells with DSB damage 0.5 h after irradiation for all irradiation geometries, as seen in 53BP1, γ-H2AX or colocalized foci per cell (Supplementary Figure S1). Generally, there were more 53BP1 DSB foci than γ-H2AX foci per nucleus. This resulted in most γ-H2AX foci (60%) colocalizing with 53BP1, while only 20% of the 53BP1 foci showed colocalization with γ-H2AX. Colocalizing foci were often restricted to the basal layer of the models or to the margins of the 66 µm high dose peaks (Figure 4), suggesting that the terminally differentiating G1 keratinocytes in the models will predominantly rely on an NHEJ/53BP1-based DNA damage response.

The average foci values per cell were highest for the 920 µm wide-field and 408 µm irradiated models (Supplementary Figure S1). For all irradiation geometries, the average damage load gradually decreased with time to reach the sham values at 72 h, with the 66 µm pMBRT being similar to sham from 6 h to 72 h. (Figure 5, Supplementary Figure S1).

### 3.3. Spatial Distribution of DNA Damage across Epidermis Models

**Figure 6:**
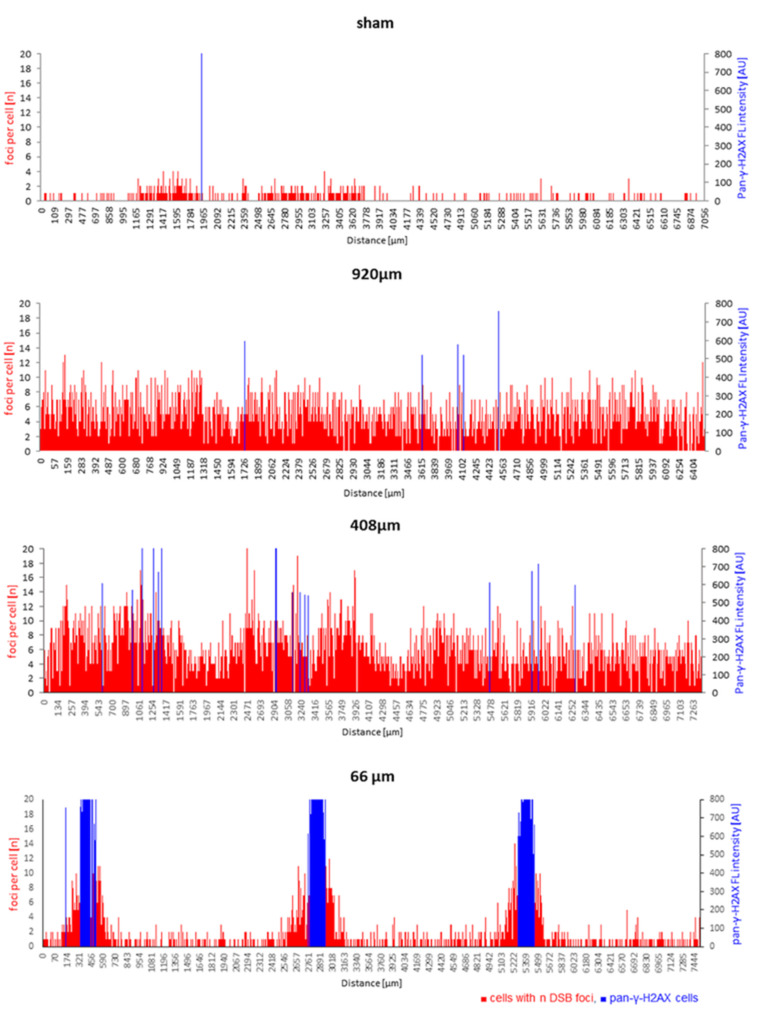
Spatial DNA damage distribution responds to beam width across proton minibeam-irradiated epidermides. The positions of cells with their respective DSB focus number (red bars) are shown along the length of the respective tissue cross-section. The height of each red bar corresponds to the number of foci within a spatially annotated cell, while the height of the blue bars indicates the position of each pan-γ-H2AX cell nucleus and its fluorescence intensity (arbitrary intensity units, AU) along a model cross-section. Sham irradiation: There is a low density of nuclei with 53BP1 DSB foci across the tissue, with most such cells having one or two DSB foci. Wide-field minibeam irradiation (920 µm): all cells show several DSB foci (up to 12) per nucleus; 408 µm minibeam irradiation: nearly all cells display DSB damage foci with regional fluctuations, while a few blue pan-γ-H2AX cells are seen; 66 µm minibeam irradiation: sharply localized clusters of cells displaying pan-γ-H2AX nuclei at the dose peaks are flanked by cells showing elevated DSB foci numbers. The distance of the peaks of pan-γ-H2AX cells corresponds to the inter-beam distances of 2500 µm. Damage in the interstitial tissue corresponds to sham-irradiated controls.

Since the average DSB values across the different models are only a crude measure, and because it is of particular interest whether proton minibeam irradiation spares non-irradiated tissue and cells at the surface between the beams, we next performed spatially resolved cell-specific DNA damage analysis along tissue model cross-sections. Since processing for paraffin embedding of the membrane-anchored tissues led to different degrees of bending/curling of the models, software-based linear alignment of position-annotated nuclei was performed (see Section 2.5). This analysis showed that DNA damage distribution and load varies with beam width and doses across the epidermides exposed to pMBRT (Figure 6).

Sham-irradiated epidermis models had cells with DSB damage distributed randomly across the tissue, with most DSB-positive cells displaying only one or two 53BP1 or γ-H2AX foci (Figure 6). In contrast, cells with DSB foci after 920 µm wide-field irradiation were uniformly distributed in the epidermis cross-sections (Figure 6). The spatial distribution of cells with different DSB damage foci numbers and cells with saturated DNA damage response, as indicated by pan-γ-H2AX staining, varied along the length of the tissue cross-sections in models irradiated with 408 µm minibeams (Figure 6). The fluctuations of foci per cell values over sub-regions of the 408 µm epidermis models did not obviously correlate with the minibeam peak dose positions, which may point to other influences, like bystander effects [34,35] or technical ones (embedding/orientation variables). On the other hand, 66 µm minibeam irradiation led to sharply localized damage hotspots at the dose peaks, as characterized by clustered pan-γ-H2AX cells flanked with cells showing increased frequencies of DSB damage foci (Figure 6). The distance of the clusters of pan-γ-H2AX cells corresponds to the 2.5 mm center-to-center distances of the 66 µm minibeam channels. Cells between the beam peaks displayed DNA damage similar to control (Figure 6), indicating sparing of the non-irradiated intermediate tissue and agrees with the zero-dose calculation of Figure 2.

**Figure 7:**
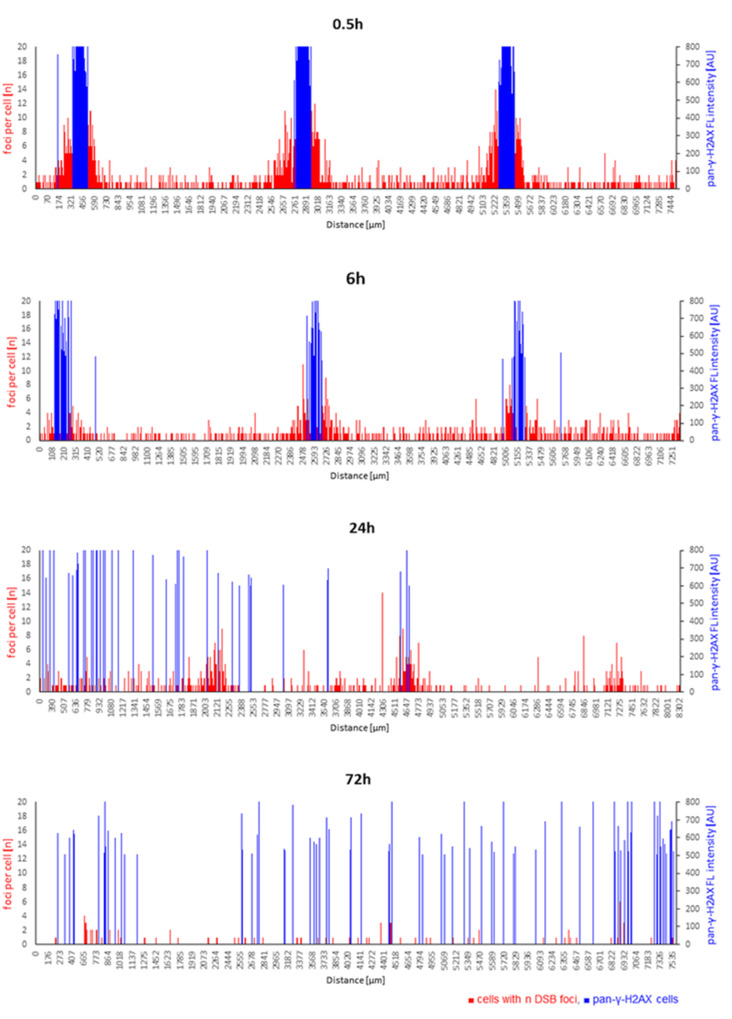
Time course of spatial DNA damage distribution and repair after 66 µm pMBRT. Cells with n DSB foci per individual cell (red bars) along the length of the tissue cross-sections, with the height of each red bar corresponding to the number of foci within a spatially annotated cell nucleus. The height of the blue bars indicates the position of a pan-γ-H2AX cell nucleus and its fluorescence intensity (arbitrary intensity units, AU) along cross-sections. pMBRT of 66 µm led to sharply localized damage at the dose peaks characterized by clustered pan-γ-H2AX cells (blue) flanked with cells showing elevated DSB foci numbers 0.5 and 6 h after irradiation. This spatial arrangement was absent at 24 h, while at 72 h of DNA repair, pan-γ-H2AX cells were scattered over the models. For comparison, a spatially annotated time course of sham-irradiated models is shown in Supplementary Figure S4.

**Figure 8:**
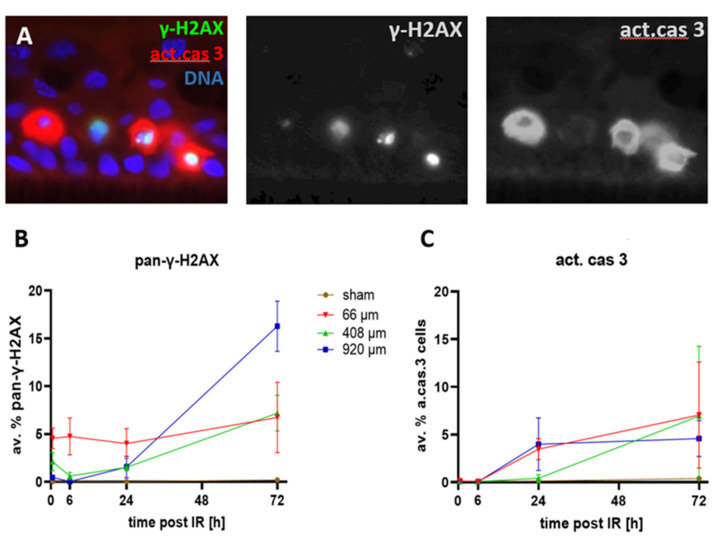
(**A**) Detail of an epidermis model showing active caspase-3 positive cells (red) contain pan-γ-H2AX-positive nuclei (green/yellowish) with nuclear fragments or pycnotic nuclei (left nucleus). Nuclear DNA: blue. Grayscale images of green and red channels to the right. The basal cell layer lies parallel to the lower edge of the image. (**B**) Frequency of pan-γ-H2AX cells over the time course. Sham-irradiated epidermides rarely displayed pan-γ-H2AX nuclei at all time points, ranging from an average of 0.07 to 0.4%. Wide-field (920 µm) pMBRT induced a significant increase (*p* < 0.05) of pan-γ-H2AX cells after 24 and 72 h. 408 µm pMBRT induced elevated levels of pan-γ-H2AX-cells relative to sham 0.5–72 h post-IR. pMBRT of 66 µm induced a significant increase of pan-γ-H2AX cells at all time points. (**C**) The average frequency of active caspase 3-positive cells in sham-irradiated tissue models was below 0.22% over the entire time course. pMBRT of 920 µm and 66 µm induced a significant increase (*p* < 0.05) over sham 24 and 72 h post-irradiation, while for 408 µm pMBRT, this was seen only at 72 h. Each data point represents the average ± SD of *n* = 9 sections of 3 independent epidermis models for each condition, except for 24 h sham (*n* = 6) and irradiated models at 72 h (*n* = 6). For data and *p*-values, see Supplementary Tables S2 and S3.

The progression of DNA repair was characterized by the disappearance of the clusters of pan-γ-H2AX cells 24 and 72 h after irradiation (Figure 7), indicating the repair of the massive DNA damage in the cells. At these two late time points, pan-γ-H2AX cells appeared that were scattered over the epidermides (Figure 7) and were more frequent in irradiated models relative to sham (Figure 8B, Supplementary Figure S4). 

### 3.4. Cell Death after Proton Minibeam Irradiation

Previous analyses have shown that minibeam irradiations with X-rays or protons lead to higher cell viabilities relative to wide-field irradiation, as assayed by the MTT assay [28]. To further investigate whether focused pMBRT is associated with cell killing and to determine whether late-appearing pan-γ-H2AX-positive cell nuclei mark cells that undergo cell death, we investigated the spatial and temporal occurrence of cells expressing pan-γ-H2AX and dying cells expressing the activated form of the executioner caspase 3 [36] (act. cas 3) in the epidermis models (Figure 8A).

Sham-irradiated epidermis models only rarely displayed pan-γ-H2AX-positive cells (Figure 8B, Supplementary Figure S4). Likewise, active caspase 3-positive apoptotic cells were only rarely seen up to 6 h after sham irradiation (<0.08%; Figure 8C), with later time points showing a slight elevation of the apoptosis frequency to 0.14% (±0.04 SD) at 24 h and to 0.4% (±0.27) at 72 h after sham irradiation (Figure 8C).

Heavily damaged pan-γ-H2AX cells were noted after pBMRT. The 66 µm and 408 µm minibeams induced a significant increase in the average frequency of pan-γ-H2AX cells at 0.5 and 6 h post-IR (Figure 8A; Supplementary Table S2). Six hours after IR, all three IR geometries displayed a significantly elevated average frequency of pan-γ-H2AX cells, with a further increase at 24 h and 72 h post-IR, with the 920 µm wide-field minibeam IR inducing the highest level of pan-γ-H2AX cells (16%; Figure 8B; Supplementary Table S2) 72 h post-IR. Figure 7 shows the spatial distribution of cells carrying DSB foci and pan-γ-H2AX DNA damage and repair progression over the time course after 66 µm pMBRT. The sharply localized clusters of pan-γ-H2AX cells 0.5 and 6 h after IR disappeared ≥ 24 h, giving way to a scattered distribution of pan-γ-H2AX cells at the two late time points. For comparison, Supplementary Figure S4 shows the spatial distribution of DNA damage in sham-irradiated models over time.

Since pan-γ-H2AX nuclei also appear in apoptotic cells [32], we next studied the activated form of the execution caspase 3 in the models. Active caspase 3-positive apoptotic cells were significantly increased 24 and 72 h after 66 µm and 920 µm pMBRT relative to sham (Figure 8C, Supplementary Table S3), whereas 408 µm pMBRT induced a significant increase of apoptotic cells 72 h after IR only (Figure 8C; for data and *p*-values see Supplementary Table S1). The spatially scattered distribution of active caspase 3-positive cells 24 h and 72 h post-IR mirrored that of scattered pan-γ-H2AX cells at these time points. Active caspase 3-expressing cells have been observed to contain pan-γ-H2AX nuclei that are sometimes fragmenting, a hallmark of apoptosis [32,37]. The active caspase 3-expressing keratinocytes in the models also contained pan-γ-H2AX nuclei indicating that the cells undergo DNA and nuclear fragmentation (Figure 8A).

## 4. Discussion

Adverse side effects to normal tissue can restrict dose escalation in radiotherapy. Here, we investigated whether spatial fractionation by proton minibeam radiotherapy [5,6] spares the non-irradiated superficial tissue, such as the epidermis, from cellular damage. So far, tissue-sparing effects of pMBRT were recognized in preclinical models as reduced inflammation and fibrosis in a mouse ear model [9,10,11] and as a reduction of brain lesions in the rat [12,13,14]. The sparing of healthy tissue may be mediated by two effects, the so-called dose-volume effect and the microscopic prompt tissue repair (for review, see [8]). While the latter effect supposedly depends on accelerated tissue repair in the exposed channels of systemic models, an increase of the maximum tolerated dose has been observed when irradiated tissue volumes are decreased [15,16]. Furthermore, bystander or rescue effects on the single-cell level might play a role in DNA damage response (DDR) [17,18,34], with an active DDR being linked to cellular senescence and inflammation [38,39].

While the epidermis models used precluded the investigation of vasculature effects, we were able to study the spatial distribution of the DNA damage inflicted by pMBRT at the subcellular level in a 3D tissue context. Thereby, we obtained evidence for the tissue-sparing effect of spatial fractionation by pMBRT at the subcellular level. We interdigitated small planar irradiated regions of different widths with non-irradiated ones by applying proton minibeams with a beam size of approximately 66 µm to 1 mm, spaced over the 8 mm of the human epiCS epidermis models that adequately reflect the epidermis response to environmental stresses [20,21]. Of the planar proton minibeam widths of 66 µm, 408 µm and 920 µm applied, the 66 µm minibeams will expose only ~17% of the cells in the skin tissue, but at a 26-fold escalated dose in the peak relative to the rather homogeneous irradiation by the 920 µm broad beam. DNA damage detection across the epidermis models revealed that the sham-irradiated epidermis models hardly displayed DSB damage over the 72 h time course, except for the 6 h time point that in all models showed a slight increase in cells carrying a few 53BP1 and γ-H2AX DSB damage foci. Apoptotic cells, on the other hand, in sham-irradiated models were below 0.5% at all time points, indicating that the ephemeral increase in DSB damage at 6 h could have originated from delayed handling effects. In test experiments, we noted that shear forces before fixation could induce activation of caspase 3. It also seems possible that temperature changes during the transport from/to the incubators and handling for mounting the tissue insets into the mylar foil-covered sample holders of the SNAKE microscope stand could have increased DNA damage, possibly by mediating replication/transcription alterations or stress [40].

Minibeam irradiation, on the other hand, induced DNA damage in all cells with the 920 and 408 µm geometries, which were repaired over the 72 h time courses conducted. pMBRT, with the 66 µm minibeam induced significant DNA damage early (0.5 and 6 h) after high-dose irradiation in the tissue streaks hit by the beam, as indicated by pan-nuclear γ-H2AX staining. While such pan-γ-H2AX cells may result from nuclear fragmentation in cells undergoing cell death [32,33,41], this seems unlikely for the 0.5 and 6 h time points, as an increase of cleaved active caspase 3-positive cells was only detected in minibeam-exposed models 24 h and 72 h post-irradiation. At these late time points, a fraction of active caspase 3-positive cells also displayed pan-γ-H2AX nuclear patterns, likely induced by DNA fragmentation during programmed cell death [41,42].

Previous research suggests that the radiosensitive keratinocytes undergoing cell death are largely cycling progenitor cells [43]. The tissue sparing effect previously noted in animal models [9,10,11,12,13,14] is reflected in our models by the absence of IR-induced DNA damage in the tissue spared from irradiation between the 66 µm focused minibeams up to 6 h after exposure. Irradiation with wider minibeams of 408 and 920 µm induced strong 53BP1-positive DSB foci in all cells over the entire models, in agreement with the inter-peak valley dose of the 408 µm minibeams being above 0.1 Gy. At lower doses, the DSB damage was largely present as a strong 53BP1 foci response, which reflects the terminal differentiation of G1 keratinocytes towards cornification, a cell cycle stage that relies on NHEJ DSB repair, of which 53BP1 is a major regulator [44,45].

When comparing the dose effects to the peak doses of the minibeams, it becomes apparent that only the 66 µm minibeams elicited a strong nuclear pan-γ-H2AX signal in the cells under the dose maxima of the minibeam. Cells of the minibeam flanking regions rather displayed DSB focus formation, while cells of the tissue between the dose peaks showed only scattered DSB foci in the range of sham-irradiated control. The different induction of pan-γ-H2AX at the dose maxima of 66 µm (27 Gy) and 408 µm (4.5 Gy) minibeams obviously differentiated the DNA damage response (DDR) of the different irradiation geometries. All the nuclei hit by the dose maximum of the 66 µm minibeams displayed a saturated ATM-dependent pan-γ-H2AX signal, while this damage pattern was seen only in a few nuclei of the 408 µm minibeam-exposed models. Obviously, the dose peak difference of a factor of ~6 determined the activation of the ATM-driven DDR and H2AX phosphorylation [46,47]. At lower doses, there was a strong DSB foci response involving 53BP1, while γ-H2AX foci were often weaker in intensity. In our experiments, this was particularly evident in the cornifying cells. Previous analyses have shown that various skin models exhibit both a potent γ-H2AX and 53BP1 DDR [6,23,24,25]. Nonetheless, in comparison to other tissues, it has been observed that ATM in undamaged skin and keratinocytes displays a predominant cytoplasmic distribution, and that irradiation induces a relocation of activated ATM to the nuclei [48]. This cytoplasmic/nuclear shuttling was also observed in a series of fibroblast cell lines and proposed to correlate with radiation sensitivity [49]. In our setting, full-blown H2AX phosphorylation was seen early after high-dose exposure with the focused beams, where one possibility is that this ATM-dependent response relies on nuclear translocation or shuttling in keratinocytes.

The clusters of pan-γ-H2AX-expressing cells early after 66 µm pMBRT failed to express active caspase 3, which indicates successful DNA repair and escape from apoptosis induction by these keratinocytes. An increase of active caspase 3-expressing cells was only noted at 24 and 72 h after minibeam irradiation, while these were spatially scattered across the tissue models, and some contained pan-γ-H2AX or pycnotic nuclei. The scattered distribution of dying cells at late time points could relate to a sensitivity of our aging epidermis models to radiation-induced reactive oxygen species from water radiolysis [50] and thus bystander effects [17,18], a possibility that deserves further investigation. When compared to the in vivo results on the tissue-sparing effect in a mouse ear skin model [10,11], it seems that the low frequency of apoptosis induction noted in the focused minibeam-exposed epidermis models is tolerated in the in vivo situation.

Overall, our results show that human epidermis models are well suited to investigate subcellular damage distribution elicited by pMBRT. Our results suggest that pMBRT can be the favorable irradiation scheme in radiotherapy to prevent DNA and tissue damage in superficial tissues.

## Data Availability

The data presented in this study are available in the article and in the supplementary material.

## Supplementary Materials

The following supporting information can be downloaded at: https://www.mdpi.com/article/10.3390/cancers14061545/s1, Figure S1: Frequency of the average DSB foci per cell with increasing repair time; Figure S2: Grey scale images of Figure 3B showing the red (53BP1) and green (γ-H2AX) channel; Figure S3: Grey scale images of the Figure 4 showing red (53BP1) and green (γ-H2AX) channels; Figure S4: Time course of the dynamics of cellular DSB foci and pan-γ-H2AX cells after sham minibeam irradiation. Table S1: Average percentages (+−SD) of cells with foci (% cwf) for the different irradiation geometries; Table S2: Average percentages (+−SD) of cells showing pan-γ-H2AX positive nuclei (% pan-γ-H2AX) for the different irradiation geometries; Table S3: Average percentages (+−SD) of cells expressing active caspase 3 (% act.cas3) for the different irradiation geometries.
