# Supplementary material for: Planar Proton Minibeam Irradiation Elicits Spatially Confined DNA Damage in a Human Epidermis Model
Source: Cancers (Basel). 2022 Mar 17;14(6):1545. doi: 10.3390/cancers14061545 (PMC8946044; doi:10.3390/cancers14061545)

Supplementary material

Supplementary Figure S1. Frequencies of the average DSB foci per cell with increasing repair time.

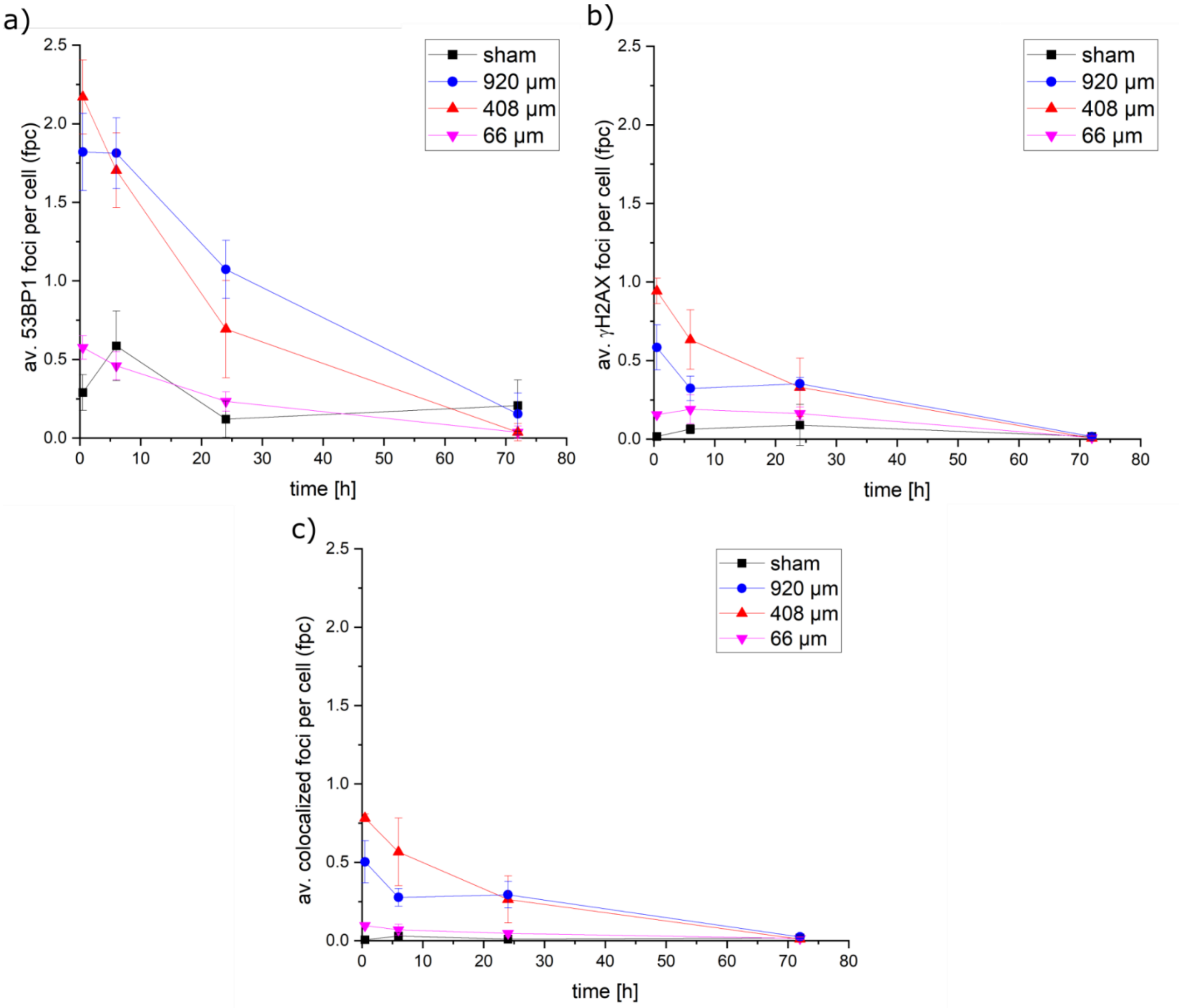

Suppl. Fig. 1: *Frequencies of the average DSB foci per cell with increasing repair time. (a) Nuclei with 53BP1 foci (foci per cell, fpc) are most abundant after 920 and 408 µm pMBRT. 66µm minibeams induced a significant difference to sham IR at 0.5 h only. (b) Time course of intense γ-H2AX foci that are less abundant in the epidermis models than 53BP1 foci. (c) Time courses of the average 53BP1+γ-H2AX colocalizing foci per cell, of which the values for 0.5h are significantly different ($p<0.05$) to sham, as are the values of 920 and 408µm minibeams up to 24h. Cells with foci reach sham values 72 h post pMBRT indicating DNA repair. In all cases, 408 and 920µm pMBRT induced the highest average DSB foci values. All average values at 72 h represent 53BP1 foci only, as large cytoplasmic background precluded analysis of γ-H2AX signals at this time point. Each data point represents the average ±SD of n=9 sections of 3 models for each condition, except for 24h sham (n=6 sections) and all irradiated models at 72h (n=6 sections).*

Supplementary Table S1. Average percentages (+-SD) of cells with foci (% cwf) for the different irradiation geometries *(see Fig. 5 for graphic display and legend)*

% cwf	0,5h	*SD*	6h	*SD*	24h	*SD*	72h	*SD*
**sham**	22.83[b]	*12.31*	42.4[a,c,d]	*10.17*	16.1[a,b]	*1.20*	15.85[b]	*11.18*
**920µm**	70.36[d]	*9.33*	73.02[d]	*4.44*	62.99[d]	*7.92*	16.19[a,b,c]	*3.46*
**408µm**	85.93[b,c,d]	*3.68*	64.1[a,c,d]	*6.48*	44.53[a,b,d]	*14.30*	4.23[a,b,c]	*4.97*
**66µm**	37.2[c,d]	*5.72*	30.4[d]	*4.39*	21.21[a,d]	*1.73*	4.42[a,b,c]	*2.92*

% cwf
a: significantly different (p ≤0.0002) to 0.5h
b: significant (p ≤0.0381) to 6h
c: significant (p ≤0.0001) to 24h
d: significant (p ≤0.0381) to 72h

% cwf	sham	*SD*	920µm	*SD*	408µm	*SD*	66µm	*SD*
**0,5h**	22.83[b,c,d]	*12.31*	70.36[a,c,d]	*9.33*	85.93[a,b,d]	*3.68*	37.2[a,b,c]	*5.72*
**6h**	42.4[b,c,d]	*10.17*	73.02[a,d]	*4.44*	64.1[a,d]	*6.48*	30.4[a,b,c]	*4.39*
**24h**	16.1[b,c]	*1.20*	62.99[a,c,d]	*7.92*	44.53[a,b,d]	*14.30*	21.21[b,c]	*1.73*
**72h**	15.85[c,d]	*11.18*	16.19[c]	*3.46*	4.23[a,b]	*4.97*	4.42[a]	*2.92*

% cwf
a: significantly different (p ≤0.0333) to sham
b: significant (p ≤0.046) to 920µm
c: significant (p ≤0.046)) to 480µm
d: significant (p ≤0.0333) to 66µm

Supplementary Table S2. Average percentages (+-SD) of cells showing pan-γ-H2AX positive nuclei (% pan-γ-H2AX) for the different irradiation geometries *(see Fig. 7B for graphic display and legend)*

% pan-γ-H2AX	0,5h	*SD*	6h	*SD*	24h	*SD*	72h	*SD*
**sham**	0.04	*0.05*	0.07	*0.12*	0.04	*0.06*	0.21	*0.30*
**920µm**	0.47[d]	*0.25*	0.04[d]	*0.04*	1.55[d]	*1.46*	16.28[d]	*2.51*
**408µm**	2.12[d]	*1.24*	0.61[d]	*0.50*	1.52[d]	*0.48*	7.20[d]	*1.78*
**66µm**	4.56[d]	*1.40*	4.75[d]	*2.50*	4,00[d]	*2.03*	6.74[a,b,c]	*3.51*

% pan-γ-H2AX
a: significantly different (p ≤0.0253) to 0,5h
b: significant (p ≤0.049) to 6h
c: significant (p ≤0.0027) to 24h
d: significant (p ≤0.049) to 72h

% pan-γ-H2AX	sham	*SD*	920µm	*SD*	408µm	*SD*	66µm	*SD*
**0,5h**	0.04[c,d]	*0.05*	0.47[d]	*0.25*	2.12[a,d]	*1.24*	4.56[a,b,c]	*1.40*
**6h**	0.07[d]	*0.12*	0.04[d]	*0.04*	0.61[d]	*0.50*	4.75[a,b,c]	*2.50*
**24h**	0.04[d]	*0.06*	1.55[d]	*1.46*	1.52[d]	*0.48*	4.00[d]	*2.03*
**72h**	0.21[b,c,d]	*0.30*	16.28[a,c,d]	*2.51*	7.20[a,b]	*1.78*	6.74[a,b]	*3.51*

% pan-γ-H2AX
a: significantly different (p ≤0.0145) to sham
b: significant (p ≤0.0026) to 920µm
c: significant (p ≤0.0145) to 480µm
d: significant (p ≤0.0028) to 66µm

Supplementary Table S3. Average percentages (+-SD) of cells expressing active caspase 3 (% act.cas3) for the different irradiation geometries *(see Fig. 7C for graphic display and legend)*

% act.cas3	0,5h	*SD*	6h	*SD*	24h	*SD*	72h	*SD*
**sham**	0.07	*0.09*	0.07	*0.07*	0.14	*0.08*	0.39	*0.27*
**920µm**	0.10[c,d]	*0.07*	0.07[c,d]	*0.10*	4.00[a,b]	*3.60*	4.59[a,b]	*1.80*
**408µm**	0.12[d]	*0.13*	0.09[d]	*0.09*	0.42[d]	*0.50*	6.95[a,b,c]	*2.95*
**66µm**	0.17[b]	*0.12*	0.09[a]	*0.06*	3.47	*1.44*	7.05	*5.30*

% act.cas 3
a: significantly different (p ≤0.0002) to 0.5h
b: significant (p ≤0.0001) to 6h
c: significant (p ≤0.0003) to 24h
d: significant (p ≤0.0003) to 72h

% act.cas3	sham	*SD*	920µm	*SD*	408µm	*SD*	66µm	*SD*
**0,5h**	0.17	*0.09*	0.12	*0.07*	0.10	*0.13*	0.07	*0.12*
**6h**	0.09	*0.07*	0.09	*0.10*	0.07	*0.09*	0.07	*0.06*
**24h**	3.47[b,d]	*0.08*	0.42[a,c]	*3.60*	4.00[b,d]	*0.50*	0.14[a,c]	*1.44*
**72h**	7.05[b,c,d]	*0.27*	6.95[a,d]	*1.80*	4.59[a]	*2.95*	0.39[a,b]	*5.30*

% act.cas 3
a: significantly different (p ≤0.0009) to sham
b: significant (p ≤0.048) to 920µm
c: significant (p ≤0.0007) to 480µm
d: significant (p ≤0.048) to 66µm

Supplementary Figure S2. Grey scale images of Figure 3B showing the red (53BP1) and green (γ-H2AX) channel.

red channel (53BP1)

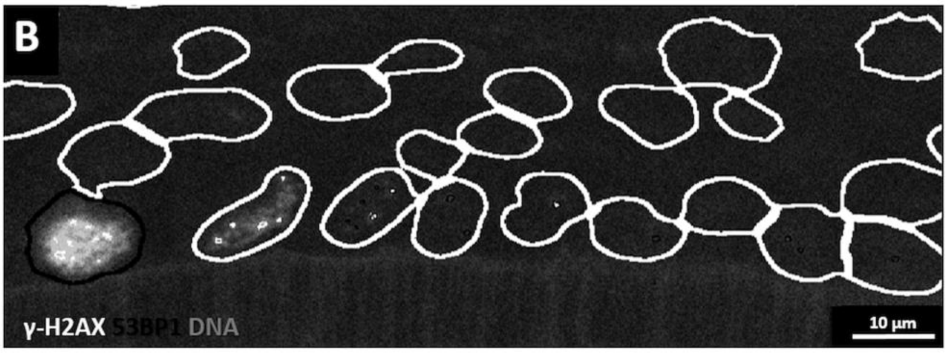

green channel (γ-H2AX)

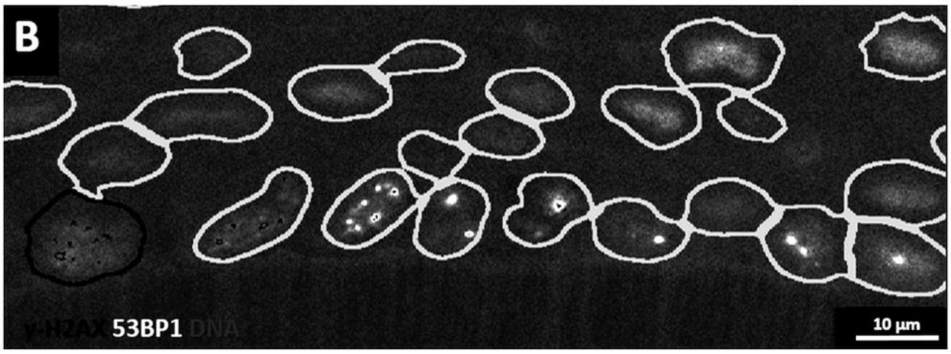

Supplementary Figure S3. Grey scale images of the Figure 4 showing red (53BP1) and green (γ-H2AX) channels.

red channel (53BP1)

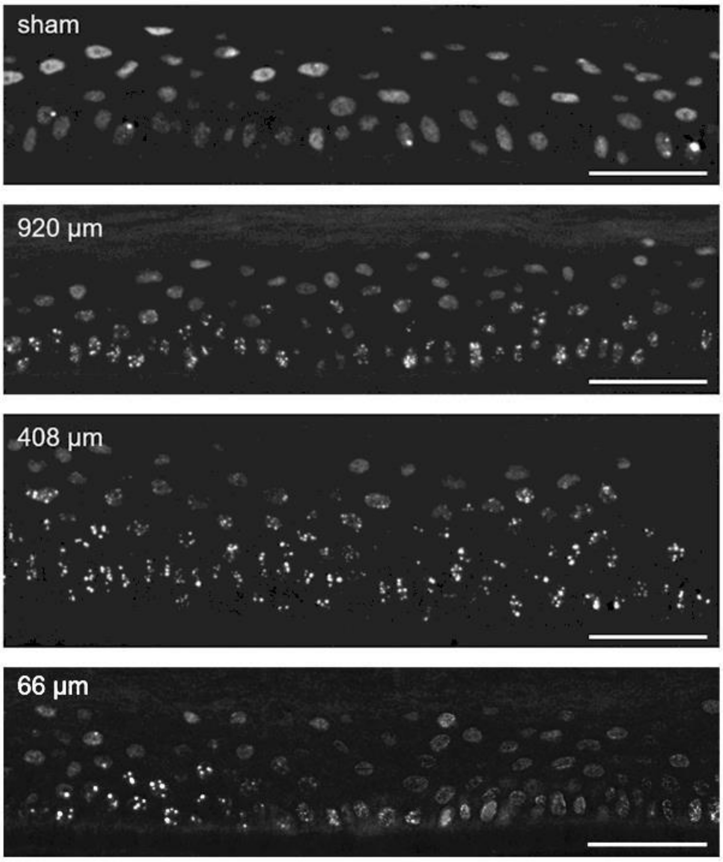

green channel (γ-H2AX)

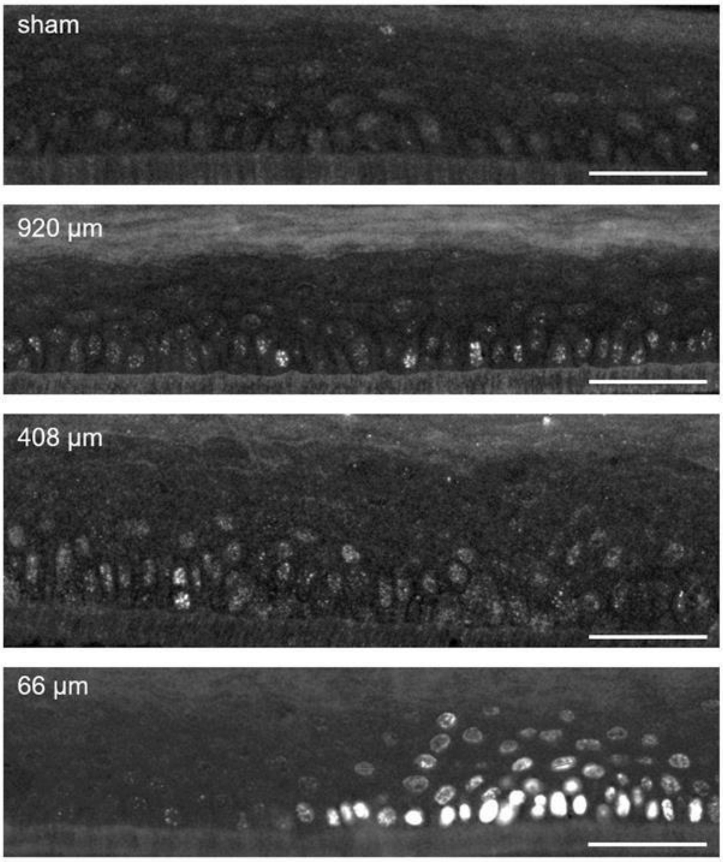

Supplementary Figure S4. Time course of the dynamics of cellular DSB foci and pan-γ-H2AX cells after sham minibeam irradiation. For legend see figure 7.

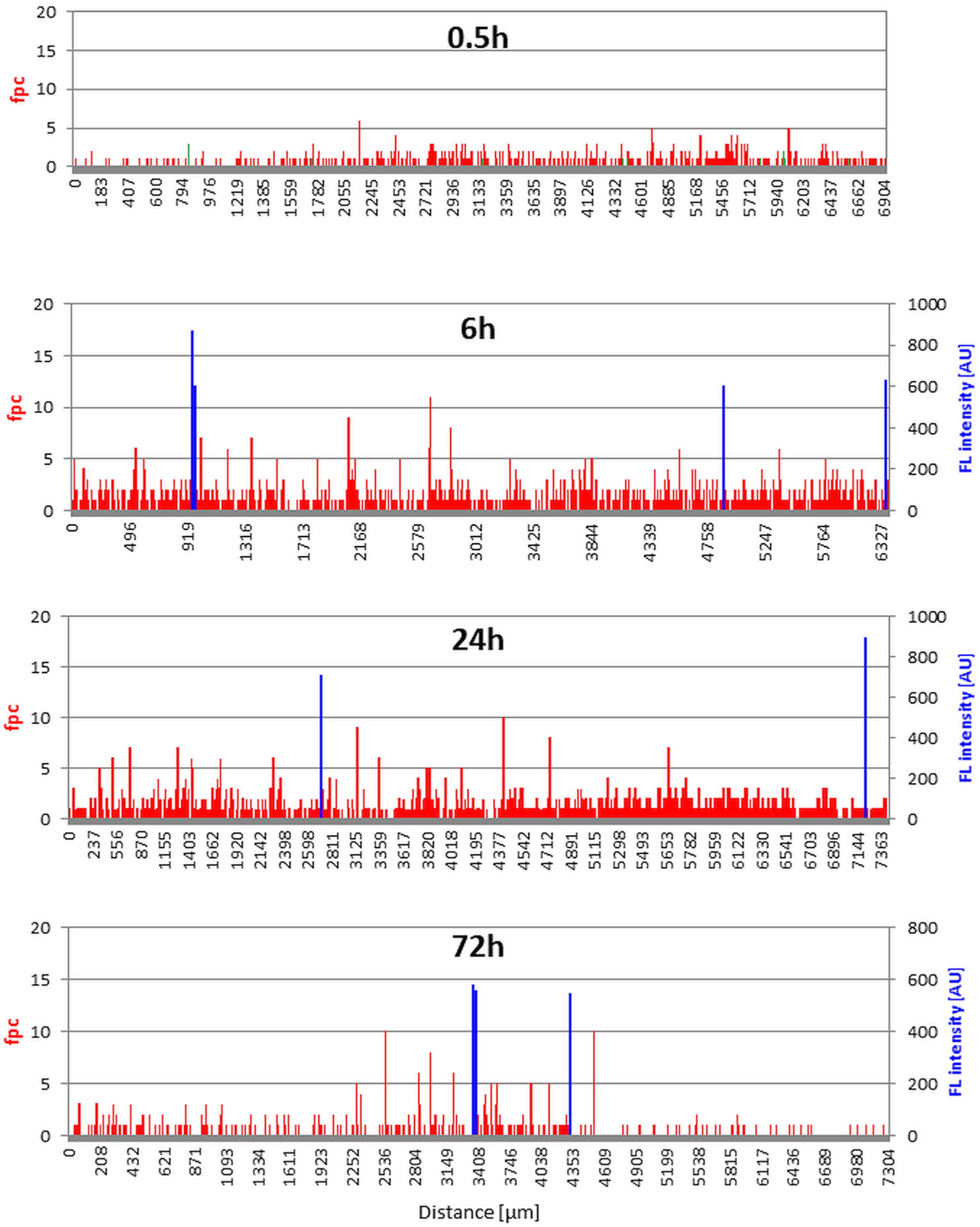

Supplement: Supplementary file 1 [file cancers-14-01545-s001.zip › cancers-1586364-supplementary.pdf]
